# Supplementary figures and images for: A high-resolution analysis of arrestin2 interactions responsible for CCR5 endocytosis
Source: eLife. 2026 Jan 19;14:RP106839. doi: 10.7554/eLife.106839 (PMC12815460; doi:10.7554/eLife.106839)

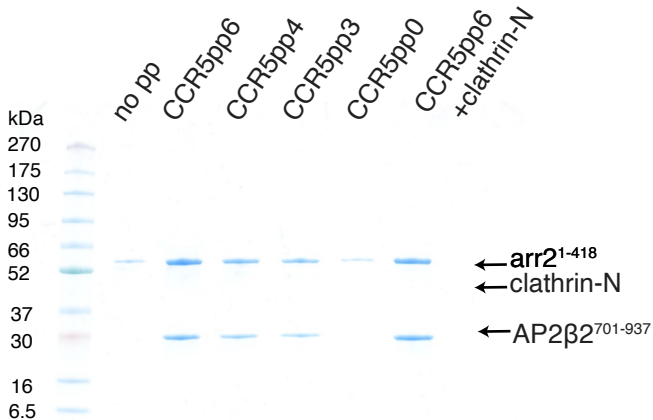

Supplement: Figure 3—figure supplement 2—source data 1. [file elife-106839-fig3-figsupp2-data1.zip › FIGURE_3_FIGURE_SUPPLEMENT_2_SOURCE_DATA_1/FigureS5_v10.pdf]

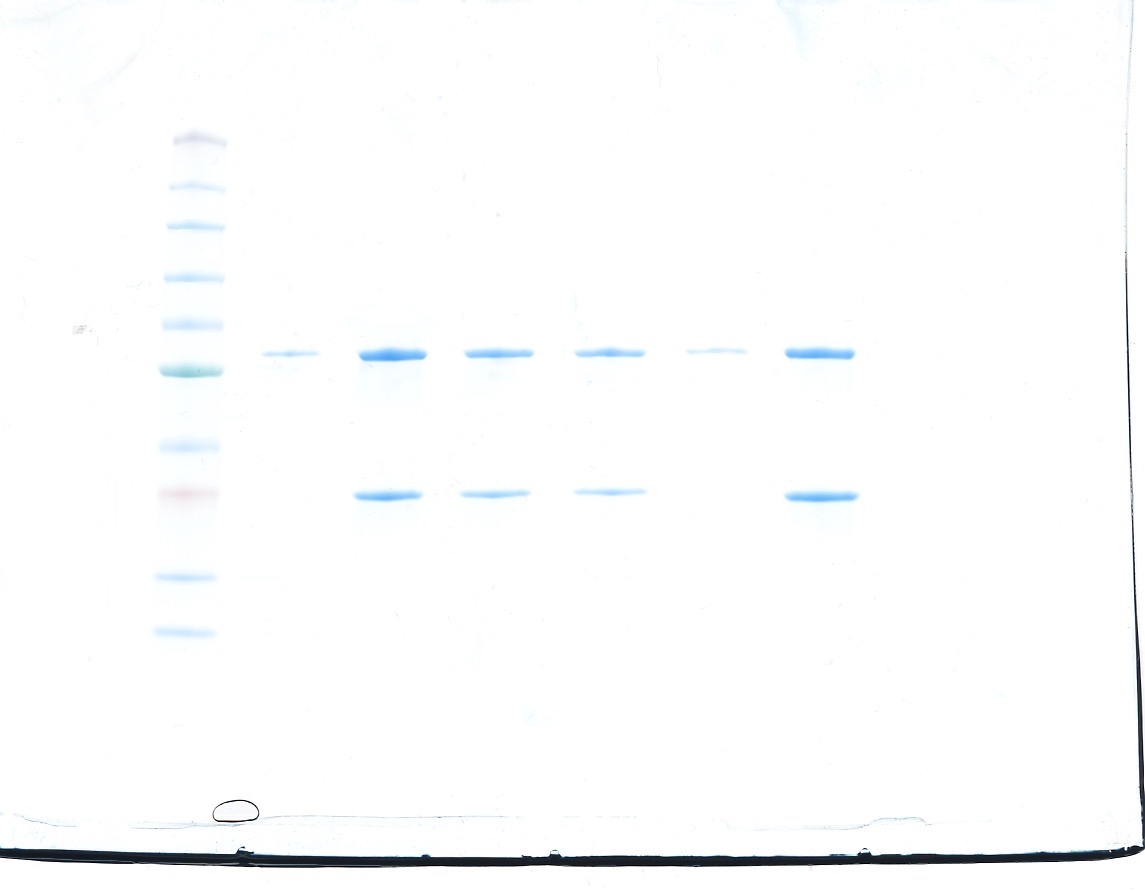

Supplement: Figure 3—figure supplement 2—source data 2. [file elife-106839-fig3-figsupp2-data2.zip › FIGURE_3_FIGURE_SUPPLEMENT_2_SOURCE_DATA_2/240628_AP2B1_arr2_SEC.jpeg]

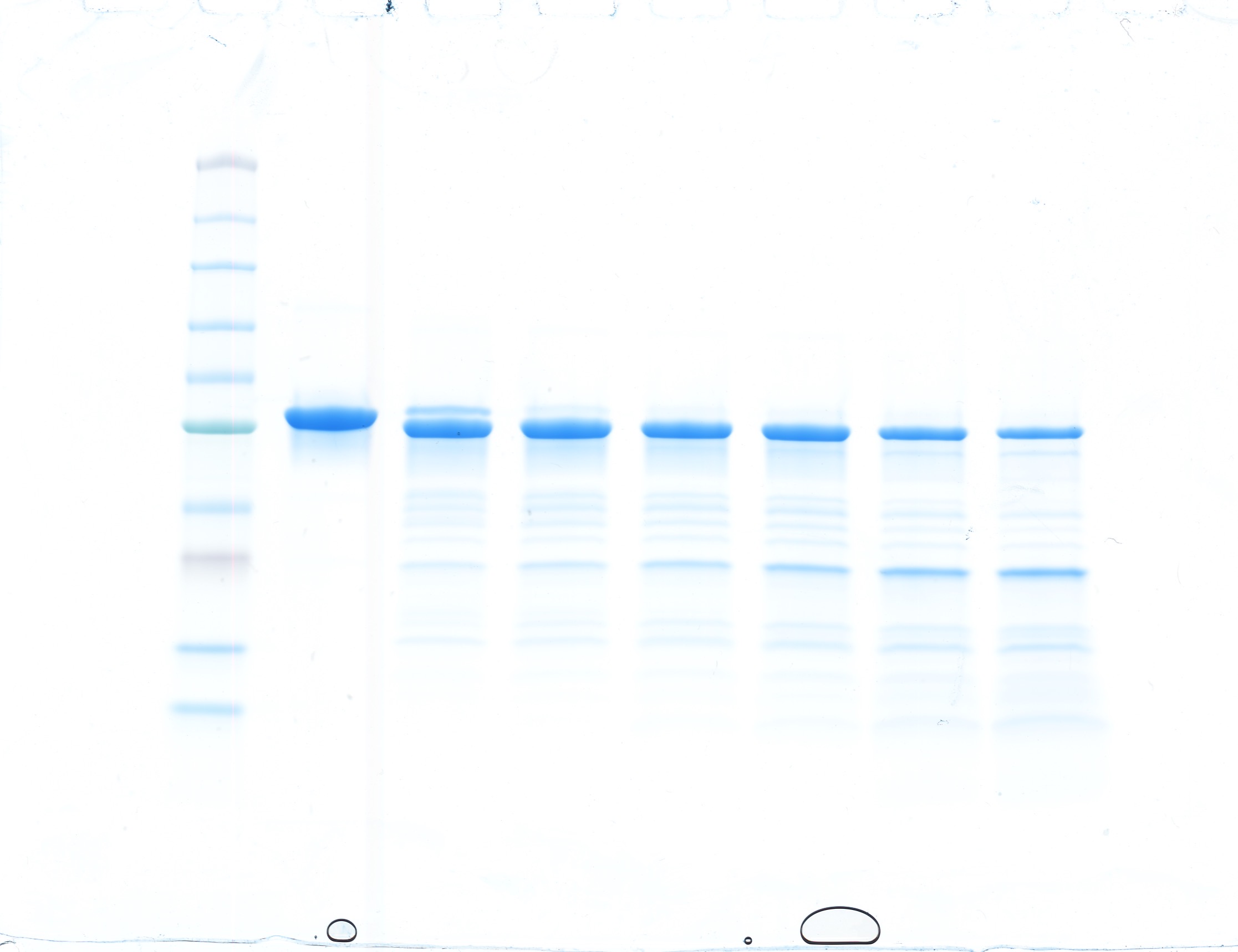

Supplement: Figure 3—figure supplement 3—source data 2. [file elife-106839-fig3-figsupp3-data2.zip › FIGURE_3_FIGURE_SUPPLEMENT_3_SOURCE_DATA_2/Panel_B_1x_arr2_25x_CCR5pp3.jpeg]

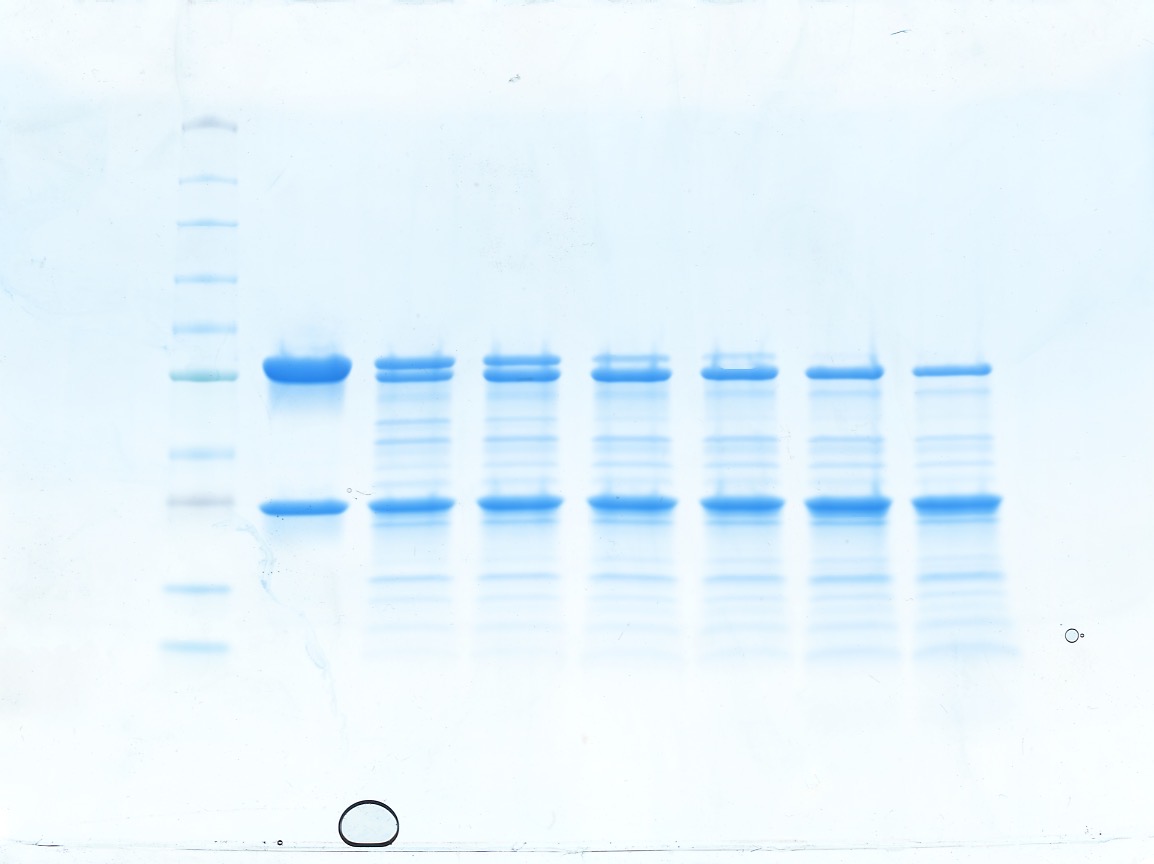

Supplement: Figure 3—figure supplement 3—source data 2. [file elife-106839-fig3-figsupp3-data2.zip › FIGURE_3_FIGURE_SUPPLEMENT_3_SOURCE_DATA_2/Panel_A_1x_arr2_5x_CCR5pp6_1x_AP2.jpeg]

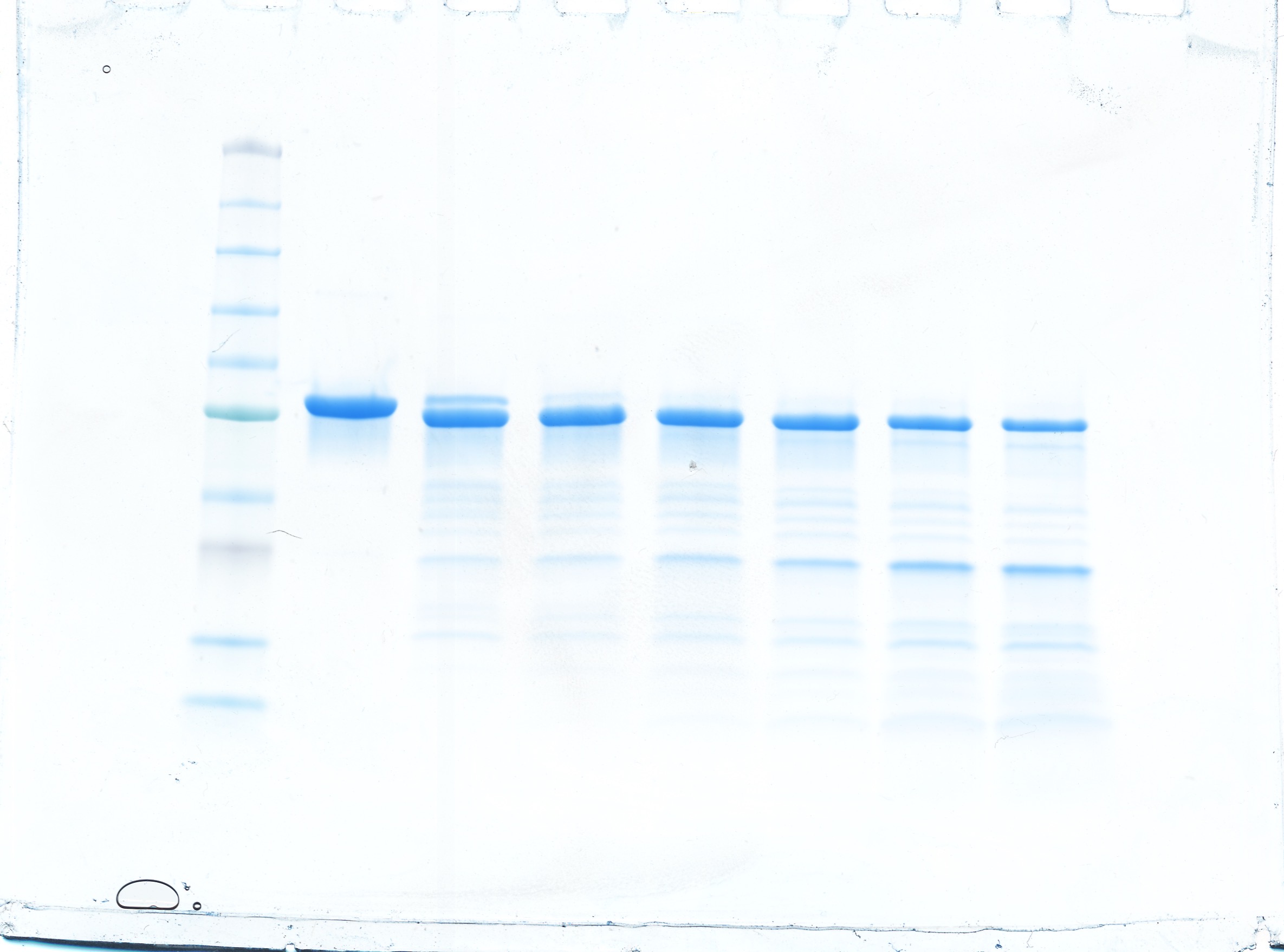

Supplement: Figure 3—figure supplement 3—source data 2. [file elife-106839-fig3-figsupp3-data2.zip › FIGURE_3_FIGURE_SUPPLEMENT_3_SOURCE_DATA_2/Panel_B_1x_arr2_25x_CCR5pp4.jpeg]

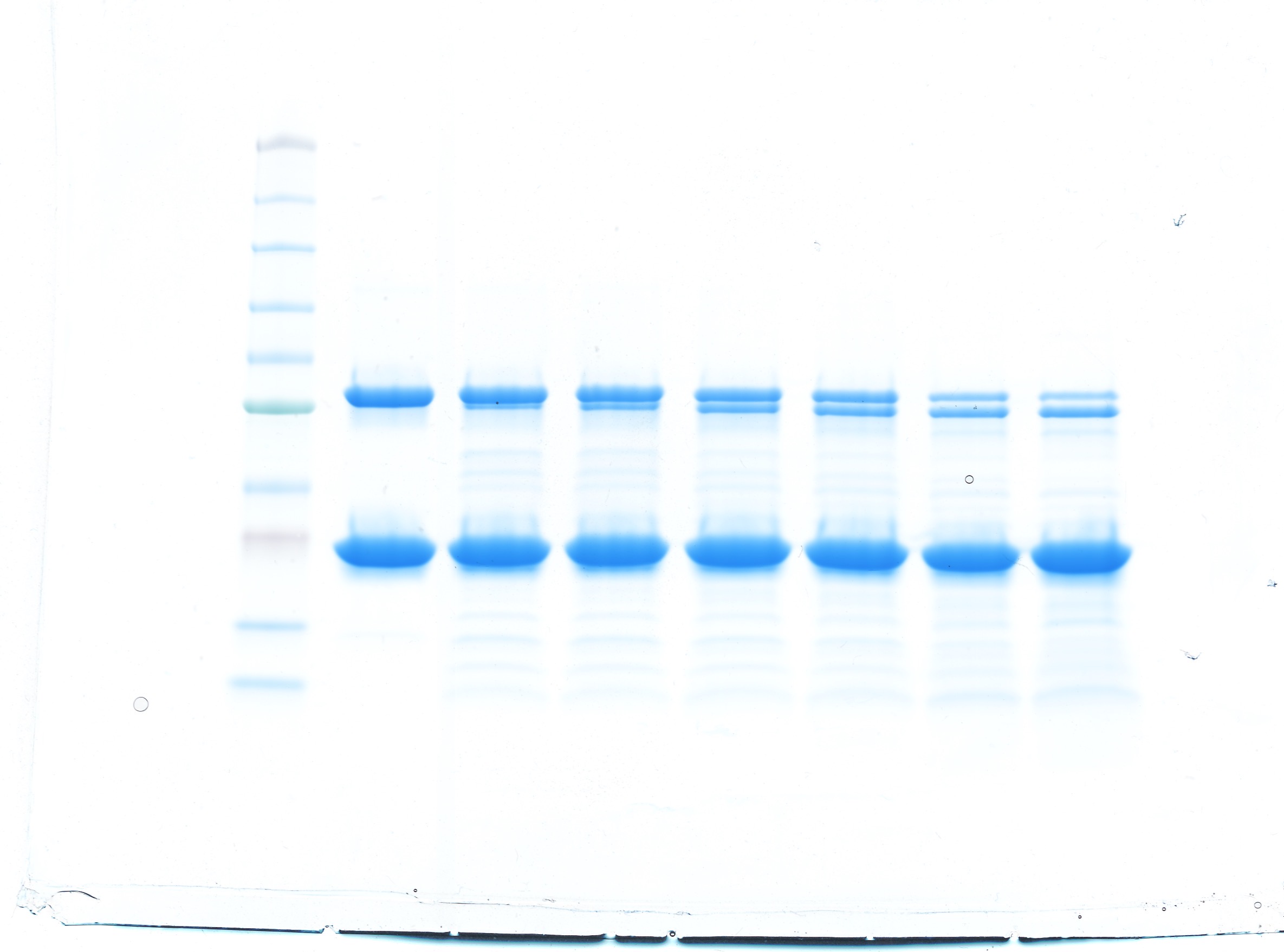

Supplement: Figure 3—figure supplement 3—source data 2. [file elife-106839-fig3-figsupp3-data2.zip › FIGURE_3_FIGURE_SUPPLEMENT_3_SOURCE_DATA_2/Panel_B_1x_arr2_25x_CCR5pp3_5x_AP2.jpeg]

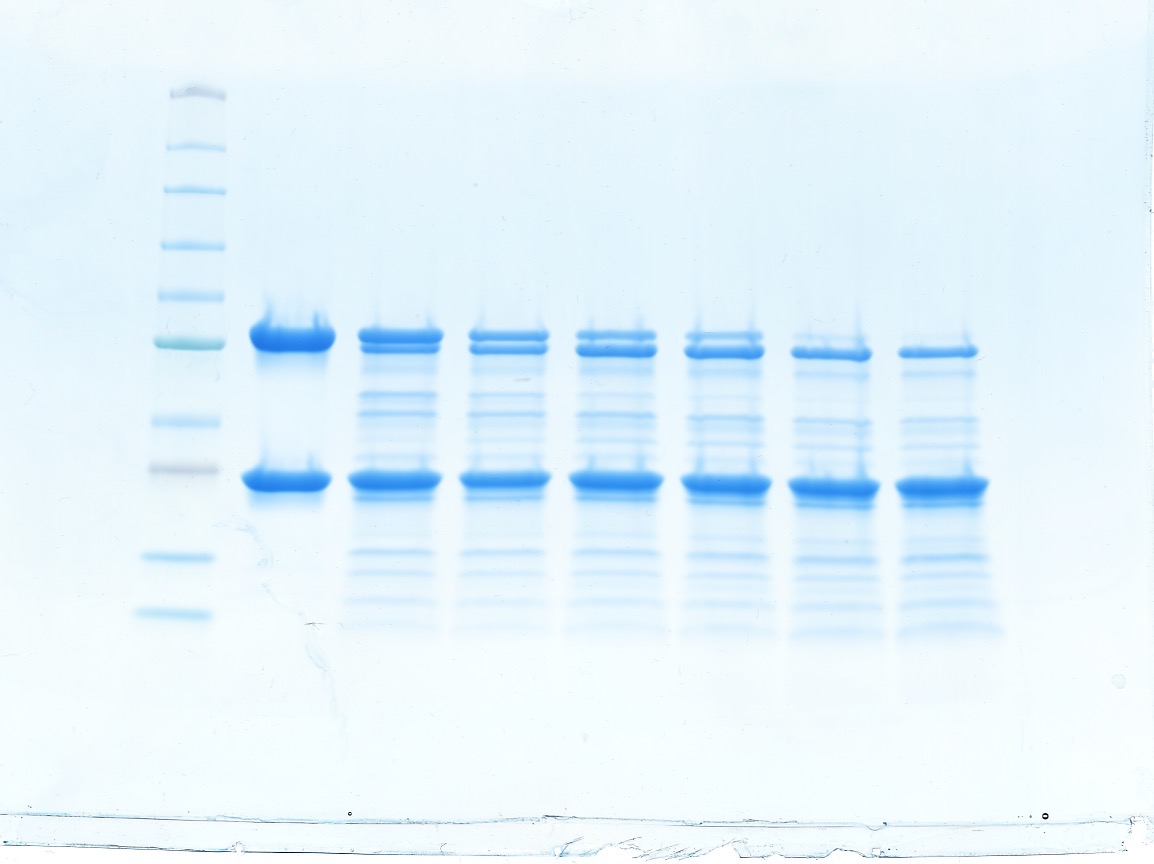

Supplement: Figure 3—figure supplement 3—source data 2. [file elife-106839-fig3-figsupp3-data2.zip › FIGURE_3_FIGURE_SUPPLEMENT_3_SOURCE_DATA_2/Panel_A_1x_arr2_5x_CCR5pp6_2x_AP2.jpeg]

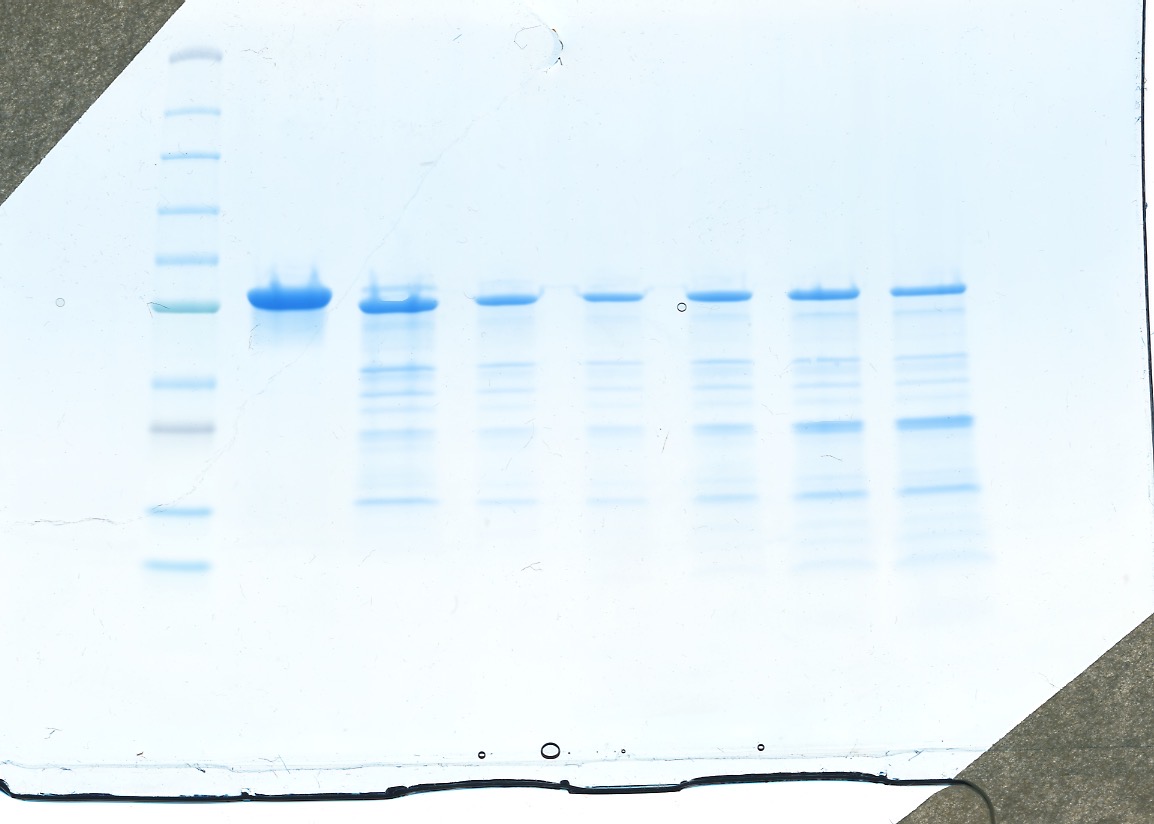

Supplement: Figure 3—figure supplement 3—source data 2. [file elife-106839-fig3-figsupp3-data2.zip › FIGURE_3_FIGURE_SUPPLEMENT_3_SOURCE_DATA_2/Panel_A_1x_arr2_5x_CCR5pp6.jpeg]

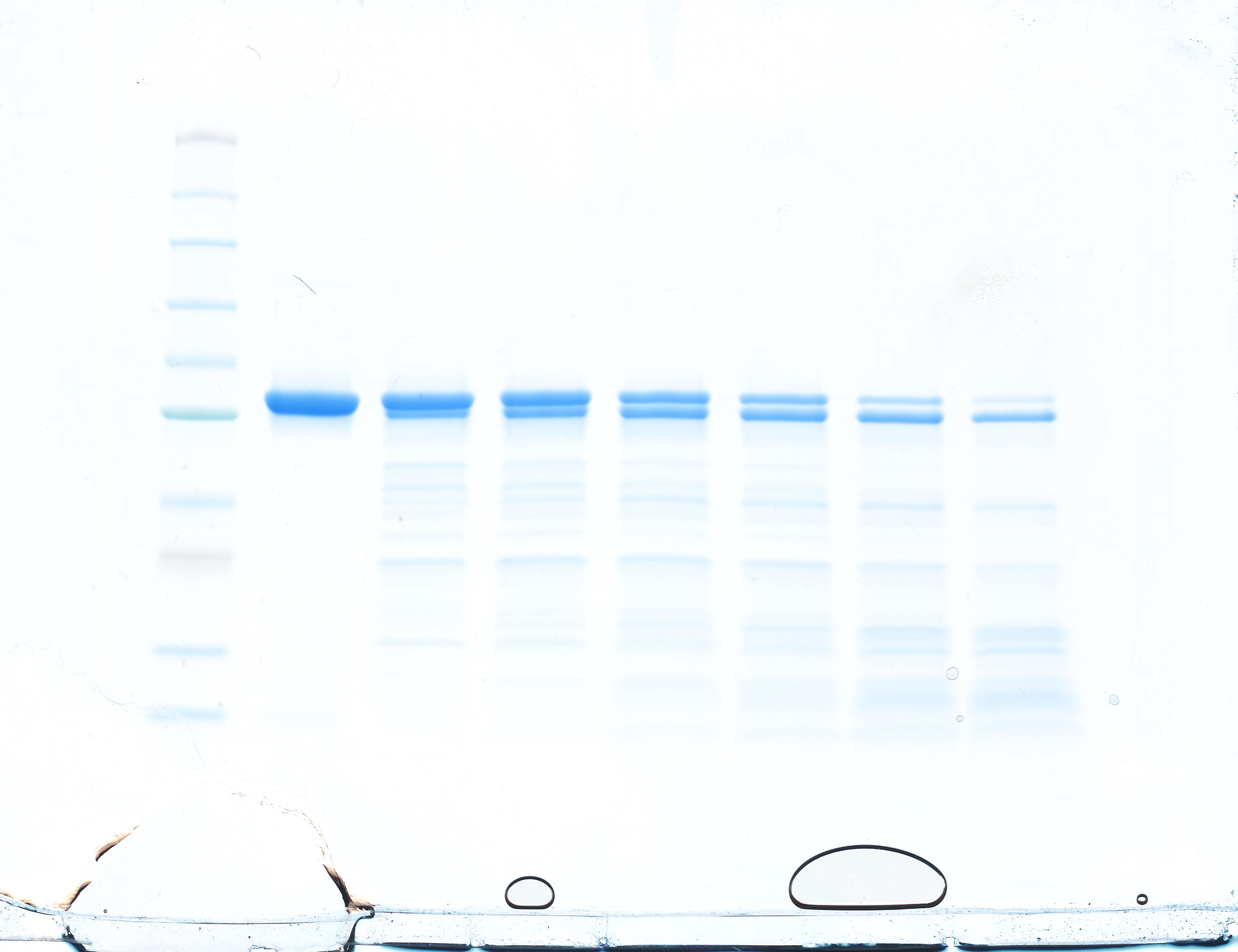

Supplement: Figure 3—figure supplement 3—source data 2. [file elife-106839-fig3-figsupp3-data2.zip › FIGURE_3_FIGURE_SUPPLEMENT_3_SOURCE_DATA_2/Panel_B_1x_arr2_3x_CCR5pp3.jpeg]

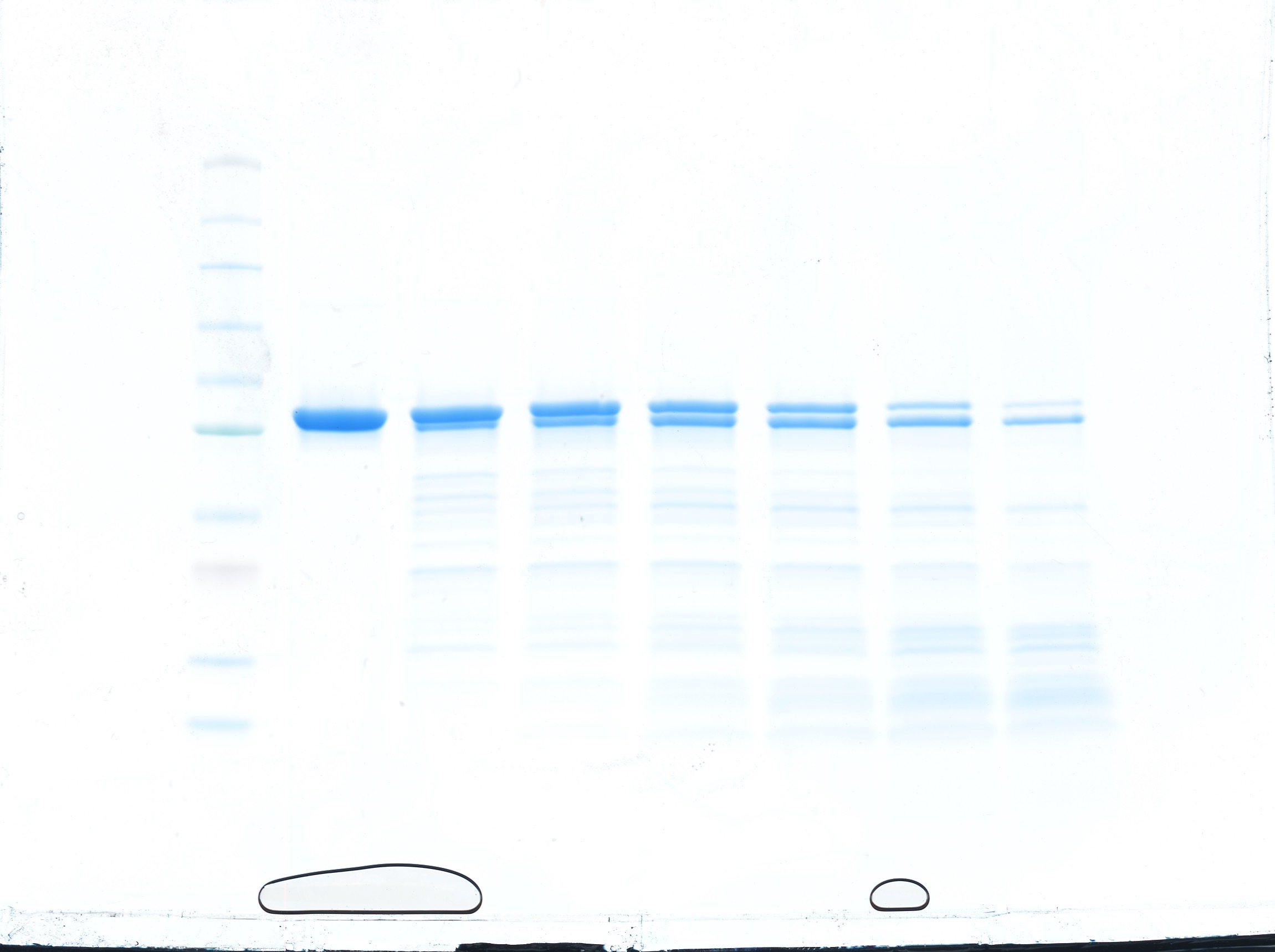

Supplement: Figure 3—figure supplement 3—source data 2. [file elife-106839-fig3-figsupp3-data2.zip › FIGURE_3_FIGURE_SUPPLEMENT_3_SOURCE_DATA_2/Panel_B_Panel_C_arr2.jpeg]

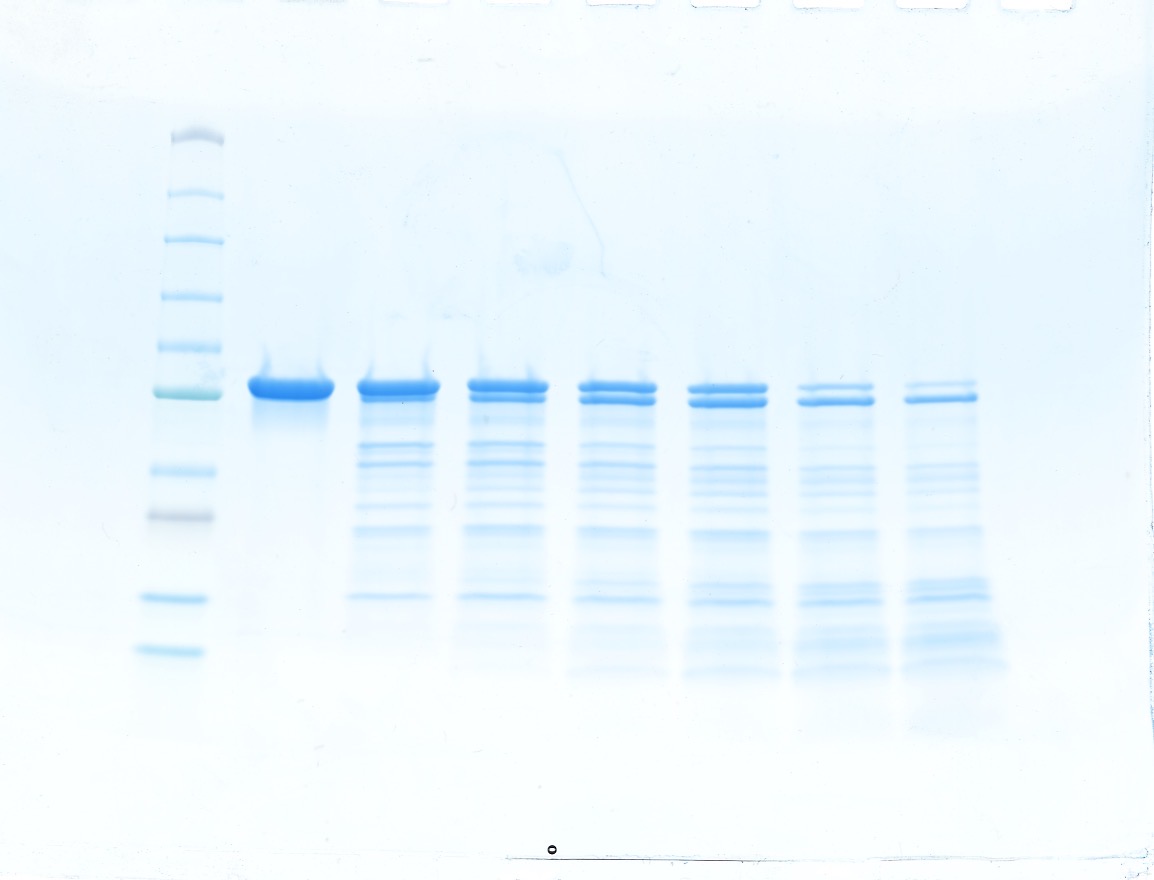

Supplement: Figure 3—figure supplement 3—source data 2. [file elife-106839-fig3-figsupp3-data2.zip › FIGURE_3_FIGURE_SUPPLEMENT_3_SOURCE_DATA_2/Panel_A_1x_arr2.jpeg]

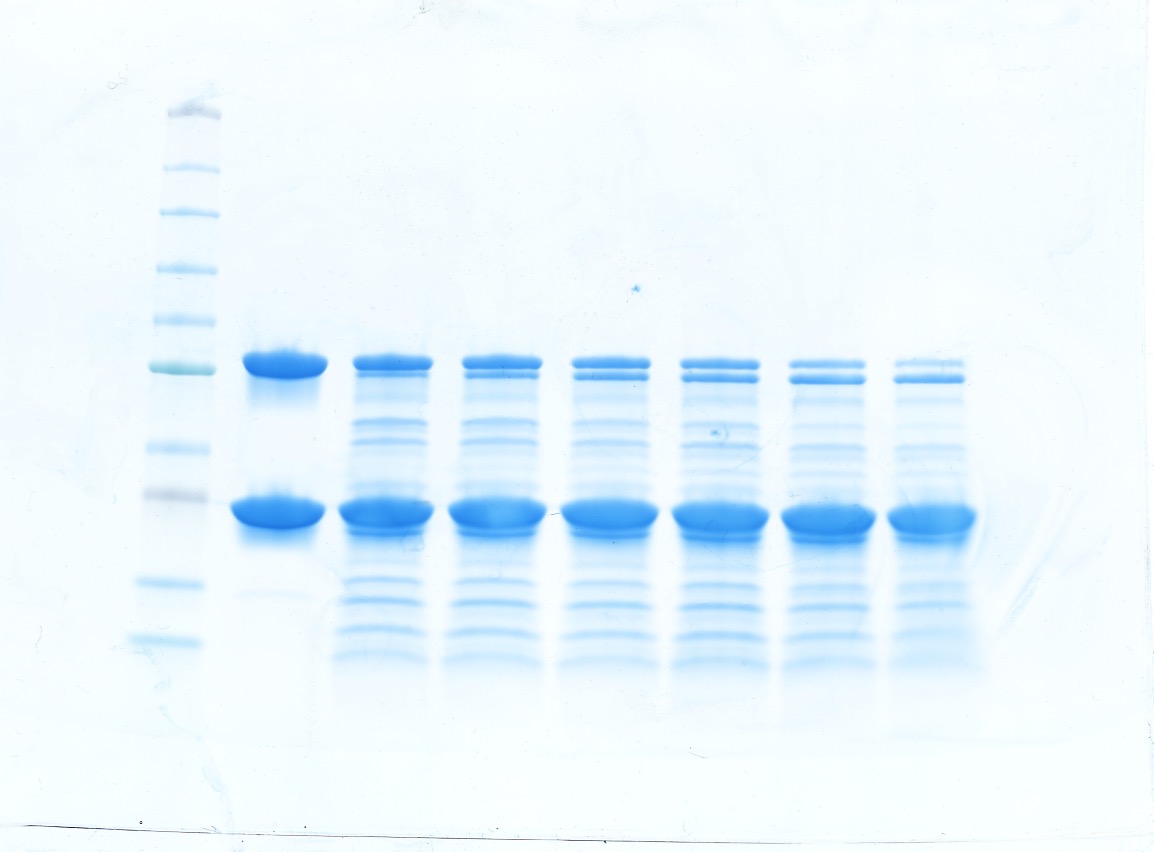

Supplement: Figure 3—figure supplement 3—source data 2. [file elife-106839-fig3-figsupp3-data2.zip › FIGURE_3_FIGURE_SUPPLEMENT_3_SOURCE_DATA_2/Panel_A_1x_arr2_5x_CCR5pp6_5x_AP2.jpeg]

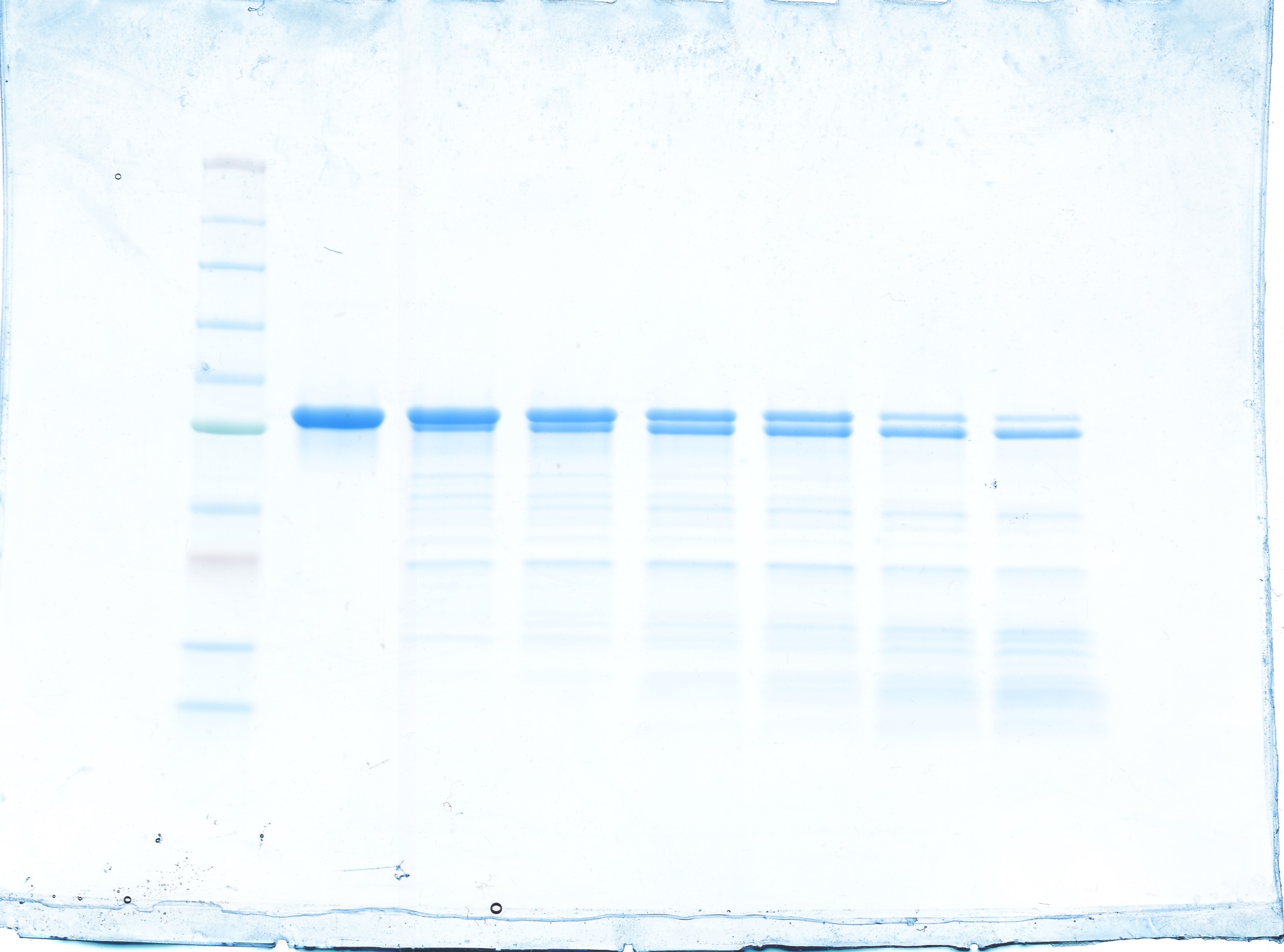

Supplement: Figure 3—figure supplement 3—source data 2. [file elife-106839-fig3-figsupp3-data2.zip › FIGURE_3_FIGURE_SUPPLEMENT_3_SOURCE_DATA_2/Panel_B_1x_arr2_3x_CCR5pp4.jpeg]

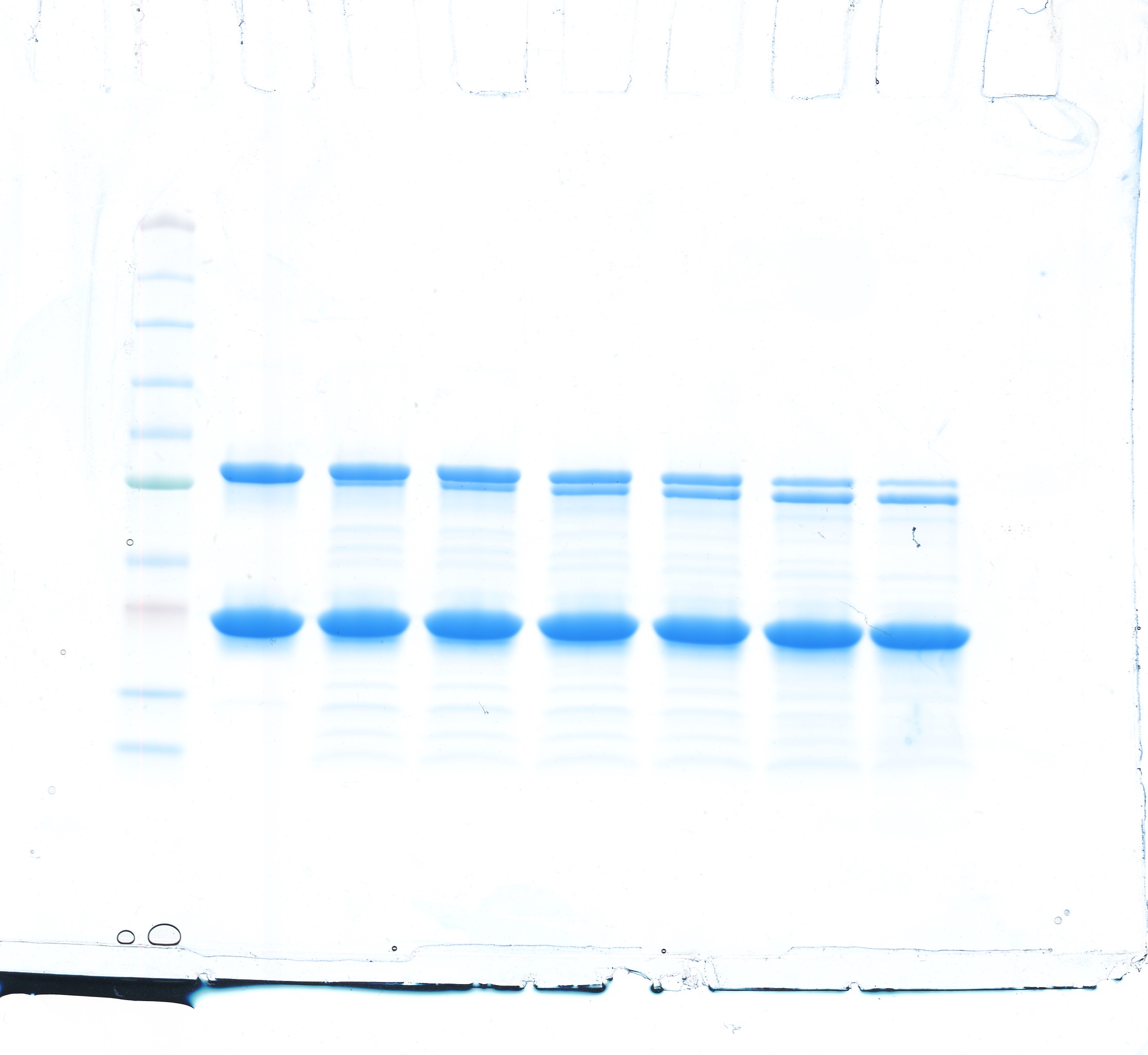

Supplement: Figure 3—figure supplement 3—source data 2. [file elife-106839-fig3-figsupp3-data2.zip › FIGURE_3_FIGURE_SUPPLEMENT_3_SOURCE_DATA_2/Panel_B_1x_arr2_3x_CCR5pp3_5x_AP2.jpeg]
