# Supplementary material for: A high-resolution analysis of arrestin2 interactions responsible for CCR5 endocytosis
Source: eLife. 2026 Jan 19;14:RP106839. doi: 10.7554/eLife.106839 (PMC12815460; doi:10.7554/eLife.106839)
Supplement: Figure 3—figure supplement 3—source data 1. [file elife-106839-fig3-figsupp3-data1.zip › FIGURE_3_FIGURE_SUPPLEMENT_3_SOURCE_DATA_1/FigureS7_v6.pdf]

for panel a

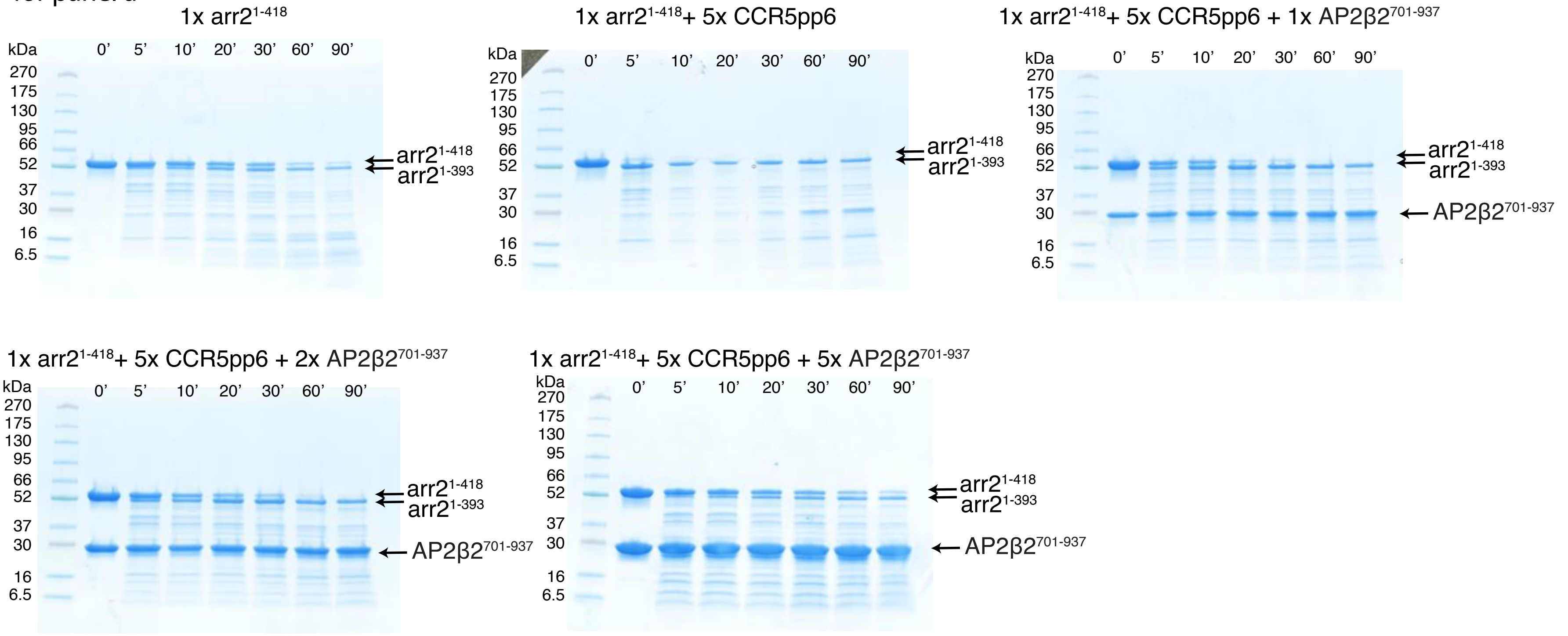

for panel b/c

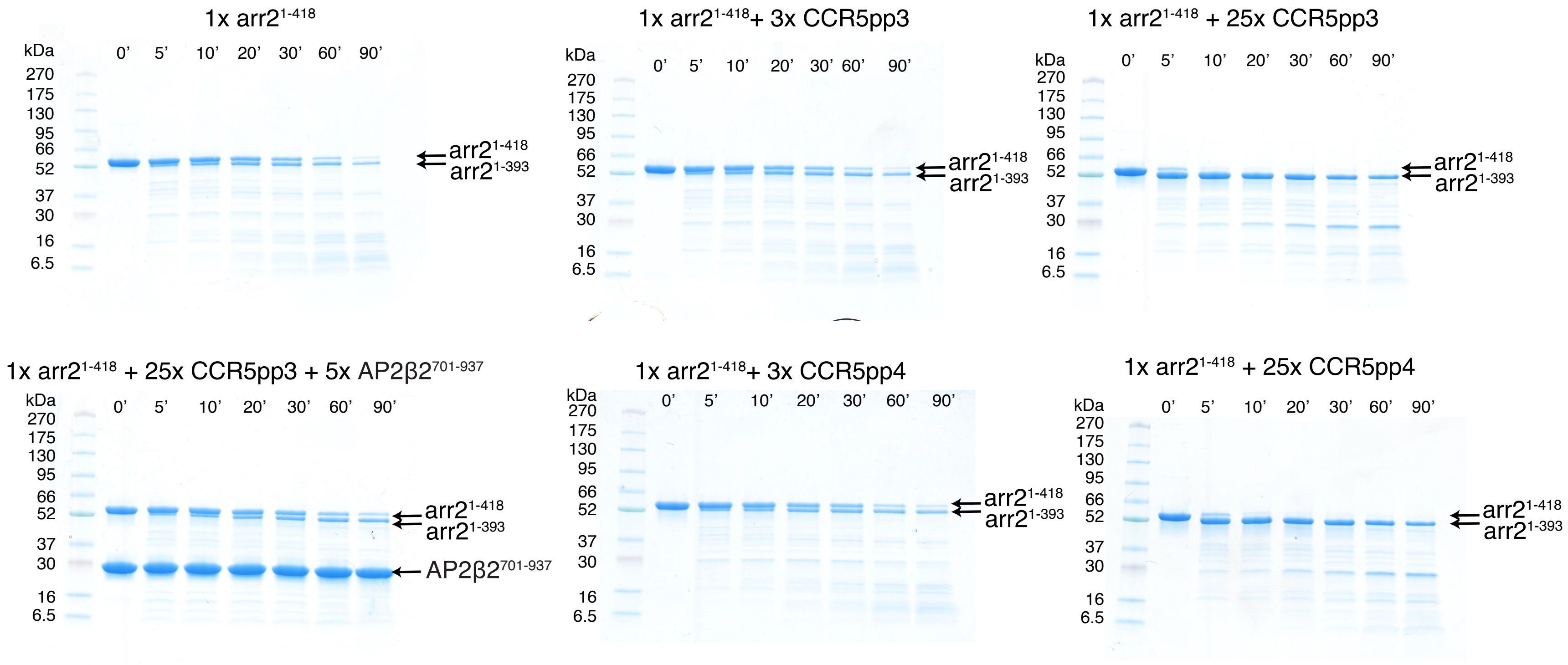

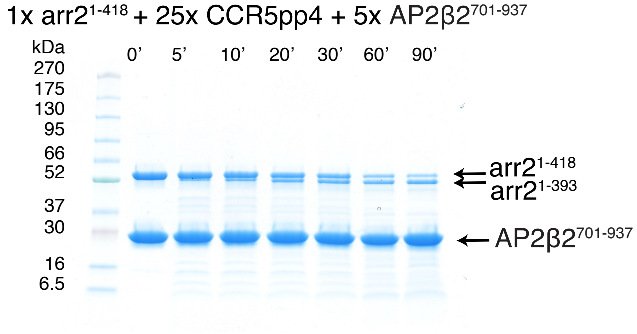

Uncropped SDS-PAGE gels of the arrestin2$^{1-418}$ trypsin proteolysis.
SDS-PAGE gels for the data in Figure 3–figure supplement 3.
The composition of the sample and the reaction incubation time are indicated at the top.
Protein marker bands are indicated on the left.
Arrestin2$^{1-418}$ runs at ~ 52 kDa, AP2β2$^{701-937}$ at ~ 30 kDa.
