# Supplementary material for: A high-resolution analysis of arrestin2 interactions responsible for CCR5 endocytosis
Source: eLife. 2026 Jan 19;14:RP106839. doi: 10.7554/eLife.106839 (PMC12815460; doi:10.7554/eLife.106839)
Supplement: Supplementary file 1. — Underlined serine or threonine residues are phosphorylated. [file elife-106839-supp1.docx]

| Phosphopeptide | Sequence | K_D_ (arrestin2^1-393^) [μM]^a^ |
| --- | --- | --- |
| CCR5pp6 | APERASSVYTRSTGEQEISVGL | 45 ± 6 |
| CCR5pp4 | APERASSVYTRSTGEQEISVGL | 199 ± 10 |
| CCR5pp3 | APERASSVYTRSTGEQEISVGL | 198 ± 6 |

^a^data from Isaikina, Petrovic et al. (2023).
